# Using a Model to Design Activity-Based Educational Experiences to Improve Cultural Competency among Graduate Students

**DOI:** 10.3390/pharmacy6020048

**Published:** 2018-06-01

**Authors:** Kathleen Bauer, Yeon Bai

**Affiliations:** Department of Nutrition and Food Studies, Montclair State University, Montclair, NJ 07043, USA; baiy@montclair.edu

**Keywords:** cultural competence, nutrition counseling, health education, multicultural education

## Abstract

To improve the cultural competency of 34 students participating in graduate nutrition counseling classes, the Campinha-Bacote Model of Cultural Competence in the Delivery of Health Care Services was used to design, implement, and evaluate counseling classes. Each assignment and activity addressed one or more of the five constructs of the model, i.e., knowledge, skill, desire, encounters, and awareness. A repeated measure ANOVA evaluated pre- and post-test cultural competence scores (Inventory for Assessing the Process of Cultural Competence among Healthcare Professionals). The overall cultural competence score significantly improved (*p* < 0.001) from “culturally aware” (68.7 at pre-test) to “culturally competent” (78.7 at post-test). Students significantly improved (*p* < 0.001) in four constructs of the model including awareness, knowledge, skill, and encounter. Factor analysis indicated that course activities accounted for 83.2% and course assignments accounted for 74.6% of the total variance of cultural competence. An activity-based counseling course encouraging self-evaluation and reflection and addressing Model constructs significantly improved the cultural competence of students. As class activities and assignments aligned well with the Campinha-Bacote Model constructs, the findings of this study can help guide health educators to design effective cultural competence training and education programs.

## 1. Introduction

Health care professionals need to learn cultural competency skills in order to provide effective care due to changing demographics [1], health care inequalities [2], regulatory agency mandates [3], increased use of complementary and traditional therapies [4], and the need to decrease the cost of health care [5]. Considerable population shifts have been occurring and are expected to continue in the United States. According to the U.S. Census Bureau, the United States has been moving toward a cultural plurality since the 1970s, where no single racial or ethnic group is a majority. By 2044, non-Hispanic whites will become less than 50% of the total population of the United States [1]. This population shift creates an array of health care challenges because each group has unique linguistic patterns, cultural characteristics, and health profiles. As of 2011, 22.4% of Americans spoke English “not well” or “not at all” [6]. In addition, there have been dramatic changes in the number of older Americans. The percentage of people 65 years of age and older is expected to climb to 24% by 2060, increasing the call for health professionals to have expertise working with individuals who have chronic diseases or disabilities and a variety of pharmaceutical needs [1].

Substantial health disparities exist based on gender, age, race or ethnicity, education, income, religion, disability, geographic location, sexual orientation, or other characteristics historically linked to discrimination or exclusion. Inequalities exist with regard to access to health care; delivery of quality, competent health care services; and health outcomes. The reasons for these inequalities are multidimensional. However, one line of attack by professional organizations, institutions of higher education, and accrediting agencies has been a focus on individual and organizational cultural competence [7]. The Accreditation Council for Pharmacy Education (ACPE) Standards 2016 mandate that pharmacy curricula prepare students to be able to provide patient-centered, culturally sensitive care to diverse groups [8].

The Office of Minority Health defined cultural and linguistic competence as “a set of congruent behaviors, attitudes, and policies that come together in a system, agency, or among professionals that enables effective work in cross-cultural situations,” drawing from Cross’s *Towards a Culturally Competent System of Care* [9,10]. In order to prepare future health care professionals to work effectively with diverse population groups, pharmacy educators advocate for creative course planning and execution [11]. The Campinha-Bacote Model of Cultural Competence in the Delivery of Health Care Services has been used to provide creative curricula guidance in a variety of health fields including pharmacy education [12]. The constructs of this model address the three cultural competency domains (knowledge, skills, and attitudes) identified as significant in medical and public health education [7]. Also, this model provides a framework for learning practical strategies to foster patient-centered communication as advocated for pharmacy best practices [13]. The Campinha-Bacote Model was used to design, implement, and evaluate nutrition counseling graduate classes focusing on improving cultural competency. The premise of this pre and post comparison study was that an interactive course exposing students to theoretical and practical components of cultural competence would be useful in improving cultural sensitivity skills. The objectives were to determine the effects of the course on the five constructs of the Campinha-Bacote Model as well as on perceived cultural competence.

## 2. Materials and Methods

The Campinha-Bacote Model of Cultural Competence in the Delivery of Health Care Services [14] looks at cultural competence as a process in which healthcare professionals continually strive to work effectively within the cultural context of a client (family, individual, or community). According to the Model, cultural competence is “a process of becoming culturally competent, not being culturally competent.” Five interdependent constructs of this model are cultural awareness, cultural knowledge, cultural skill, cultural encounter, and cultural desire. Cultural competence is influenced by working on any of these areas and strengthens the impact of the others on the journey towards cultural competence. Research of this model indicates that cultural encounters play a pivotal role in the process by having the greatest influence on the other four constructs. Specific learning experiences in the graduate classes addressed the Model’s constructs.

Cultural awareness is defined as “the deliberate self-examination and in-depth exploration of our personal biases, stereotypes, prejudices and assumptions that we hold about individuals who are different from us [14].” The goal of lessons and activities focusing on this construct was to develop awareness that cultural beliefs and values influence conscious and unconscious thoughts and to understand that these create a bias of acceptable behavior. For one learning experience, students prepared and discussed collages illustrating cultural factors influencing their perceptions of the world. In order to challenge assumptions, students participated in Barnga, a simple card game simulation in which players needed to negotiate cultural clashes based on different perceptions and rules [15]. In order to create awareness of “thoughts and feelings outside of conscious awareness and control”, students took an online test to uncover potential prejudices [16].

Cultural knowledge involves seeking and obtaining a sound educational foundation of diverse cultural groups regarding cultural values, health-related beliefs and practices, and disease incidence and prevalence. As can be seen in Table 1, a variety of assignments and activities addressed this construct in order to enhance students’ knowledge of a vast range of cultural characteristics. In particular, the learning activities focused on cultural terms, cultural competency models, ethnopharmacology (scientific study of medicinal practices of cultural groups), cultural values, health disparities, organizational cultural competence, and in-depth investigation of selected cultural groups focusing on ethnicity, disabilities, and lifespan issues.

Cultural skill is the ability to use appropriate cross-cultural communication skills to collect relevant cultural data and health histories in order to provide an appropriate and sensitive client-centered health intervention. A number of guidelines have been developed to assist health professionals in providing effective and culturally sensitive interventions. For the most part, they evolved from Arthur Klienman’s theory of explanatory models which proposes that individuals and groups can have immensely different perceptions of health and disease [33]. One guideline developed for health professionals is the 4 C’s of Culture, a mnemonic developed by Drs. Stuart Slavin, Alice Kuo, and Geri-Ann Galanti, to remember what questions to ask in order to obtain the client’s point of view. The C’s refer to the following: What do you CALL your problem? What do you think CAUSED your problem? How do you COPE with your condition? What CONCERNS do you have regarding your condition [34]? Berlin and Fowkes LEARN Model provides guidelines for cross-cultural interventions. This mnemonic refers to the following: LISTEN with empathy and understanding. EXPLAIN the recommended plan of care. ACKNOWLEDGE your patient’s understanding of his illness and how they agree or differ with the recommended plan. RECOMMEND a plan of care integrating your patient’s suggestions when possible. NEGOTIATE options on a course of action [35]. Students learned these skills through videos, readings, lectures, in-class technique practices, and working with volunteer clients to practice cross-cultural interview methods. In addition, students interviewed a counselor who worked with the cultural group chosen to investigate.

Cultural encounters with individuals from diverse cultural backgrounds encourage practitioners to appreciate alternative interpretations of reality and possibly question pre-existing beliefs about a specific cultural group. Encounters create opportunities to develop attitudes congruent with cultural competence, such as appreciation, curiosity, and respect. Throughout the course, students participated in a variety of cross-cultural encounters through readings, videos, presentations, cross-cultural interviews, and individual field trips. One component of the course was an eight-week book club. Students read *The Spirit Catches You and You Fall Down—A Hmong Child, Her American Doctors, and the Collision of Two Cultures* by Anne Fadiman, winner of the 1997 National Book Critics Circle Award for nonfiction. This was both an in- and out-of-class activity. Students read designated chapters and journaled answers to assigned questions [42]. For the duration of the book club, the first 15 min of each class began with students meeting in small groups to discuss their perceptions of the most recent reading.

Cultural desire is the motivation of healthcare professionals to engage in the process of becoming culturally competent. By valuing diversity, practitioners are more likely to provide appropriate and compassionate service and meet the needs of their clients. During the course, the importance of valuing desire was discussed. Although no activity was identified that could specifically address this construct, the assumption was made that desire would ultimately be affected because one of the tenets of the Campinha-Bacote Model is that working on any one of the five constructs influences all of them.

An overview of constructs and learning experiences can be found in Table 1.

All graduate students majoring in Nutrition and Food Science with a concentration in Nutrition Education are required to take Nutrition Counseling for Diverse Population Groups. This is a one-semester course that meets for 2.5 h once a week for 16 weeks. All of the students who took the course during the spring of 2010 and 2011 participated in the evaluation. Each class had 17 students, so there was a total of 34 participants. Twenty-seven participants were 20–29 years old, 2 were 30–39 years old, and 5 were 40 to 49 years old. Twenty-eight students were Caucasian, 5 were African American, and 1 was Asian. There were 3 male and 31 female participants.

Institutional Review Board approval was granted from Montclair State University. All students were voluntary participants and were notified of the experimental evaluation of the course. Participants signed informed consents. All data collection was done by research assistants and all evaluation forms were coded. Coded evaluation forms were made available to the instructor at the end of the course after students received their grades.

The Inventory for Assessing the Process of Cultural Competence among Healthcare Professionals-Revised (IAPCC-R), 2002, and end-of-semester student evaluations were used for quantitative measures [14], whereas personal journals and open-ended queries provided feedback regarding participant perceptions of the effectiveness of the course on gaining cultural competence and desire to engage in the process of becoming culturally competent. IAPCC-R was used to measure overall cultural competence as well as levels of competence for the five constructs of the Campinha-Bacote Model at the beginning of the semester and after completion of the course. The IAPCC-R is a pencil/paper self-assessment tool and has a Likert-type scale with 25 items. Response options ranged from 1 (strongly disagree) to 4 (strongly agree). The total scale ranges from 25 to 100. A score of 25 to 50 identifies cultural incompetence, a score of 51 to 74 indicates cultural awareness, a score of 75 to 90 specifies cultural competence, and a score of 91 to 100 designates cultural proficiency. This tool has been used extensively within the United States and has been tested for reliability and validity for health care professionals. Reliability studies conducted in 12 states yielded an average reliability coefficient Cronbach alpha of 0.83 [32]. A repeated measure ANOVA was used to analyze the cultural competency constructs of the Campinha-Bacote Model.

Because the course activities and assignments were designed to fit the Model constructs, exploratory factor analysis was performed. This analysis was used to assess the validity of the design, i.e., did the specific activities and assignment actually align with the constructs? If the design is valid, assignments and activity data (factor loadings) will gather around factors and correspond to model constructs. A factor loading of 0.55 (good) was used when extracting factors and finding commonality with specific constructs [43].

## 3. Results

Evaluation of cultural competency constructs of the Campinha-Bacote Model improved significantly after completion of the course (see Table 2). The total competence score improved (*p* < 0.001) from “culturally aware” (score of 68.7 at pre) to “culturally competent” (score of 78.7 at post). The scores for each construct of the model also improved after completion of the course. Scores for competency in awareness, knowledge, skill, and encounter all improved significantly (*p* < 0.001). Desire improved but statistical analysis did not indicate significance. Of the 34 participants, 27 improved in awareness, 28 improved in knowledge, 30 improved in skill, 21 improved in encounter, and 14 improved in desire. See Table S1 in Supplementary Materials for a complete listing of pre and post construct data for individual participants.

Factor analysis of class lecture topics and assignments revealed 5 factors and accounted for 74.6% of the total variance of cultural competence (Table 3), showing that these learning experiences were very effective in helping students improve cultural competency. Factor 1 indicated knowledge, Factor 2 represented skill, Factors 3 and 4 represented encounter, and Factor 5 addressed awareness. Interestingly, the 5 assignment factors collected corresponded with the 4 predesigned Model construct categories.

Factor analysis of the 18 class activities revealed 6 factors and accounted for 83.2% of the total variance of cultural competence (Table 4), showing that these learning experiences were also very effective in helping students improve cultural competency. This factor evaluation also corresponded with the 4 predesigned Model construct categories. Factors 1 and 2 indicated the encounter construct and knowledge; Factors 3 and 4 represented the skill construct; Factor 5 addressed encounter, skill, and knowledge; and Factor 6 signified the awareness construct. The Campinha-Bacote Model postulates that working on any of the five constructs of the model influences the others. Results of the factor analysis of class activities revealed this interrelationship.

At the end of the semester, students were queried regarding their perceptions of meeting course objectives. On a 5-point Likert scale evaluating the overall usefulness of the course “in your journey to obtain cultural competence”, 33 students indicated “very useful” or “useful”. Also using a 5-point Likert scale with 1 indicating yes and 5 designated as no, all students indicated a number 1 or 2 to the following statement: “I have a greater ability to conduct nutrition counseling across cultures.” Similarly, all students designated a 1 or 2 to a statement indicating a greater desire to work with diverse population groups and another statement regarding knowledge of various cultures. In addition, students designated a 1 or 2 to the following statement: “I have a greater understanding of my own cultural attitudes, beliefs, and values.”

## 4. Discussion

In November 2016, the board of the American Association of Colleges of Pharmacy (AACP) approved the following proclamation: “AACP affirms its commitment to foster an inclusive community and leverage diversity of thought, background, perspective and experience to advance pharmacy education and improve health” [44]. A driving force for government and professional organizations emphasizing the need to prepare health practitioners to work effectively with diverse population groups has been the inequality of healthcare in the United States. As stated in the Health and Human Services Action Plan to Reduce Racial and Ethnic Disparities: A Nation Free of Disparities in Health and Health Care [45], “The ability of the healthcare workforce to address disparities will depend on its future cultural competence and diversity.” In order to provide guidance, the United States Department of Health and Human Services (DHHS), Office of Minority Health (OMH) created National Standards for Culturally and Linguistically Appropriate Services in Health and Health Care. The principal standard states, “Provide effective, equitable, understandable, and respectful quality care and services that are responsive to diverse cultural health beliefs and practices, preferred languages, health literacy, and other communication needs” [46]. In addition, the OMH launched the Think Cultural Health website, offering continuing education programs and guidance on effective patient communication for health professionals [47].

In 2007, a paper was published providing the results of a survey distributed in the United States and Canada showing that 33 of the 49 responding colleges of pharmacy used didactic lectures as their most frequent delivery method of cultural communication, skills, and knowledge. However, 49% of the respondents indicated a need to make curricular changes [48]. Since that time a number of pharmacy education programs have explored integrating activity-based learning into their cultural competency curriculum and have met with success; however, educators continue to explore methods to “adequately equip pharmacy students with knowledge of health disparities and skills in delivering culturally competent care to diverse patient populations” [49,50,51]. Sharing of best practices for achieving cultural competence within both pharmacy education and as well as training of other health professions is encouraged [49,52].

Health educators frequently use psychosocial models to design, implement, and assess outcomes of health education interventions. That concept was uniquely applied to curriculum development for a graduate nutrition counseling course using a cultural competence model. Evaluation of pre- and post-test data showed that cultural competence improved significantly. In addition, all constructs of the model also improved; however, the desire construct did not significantly increase in value. This finding is likely due to the composition of the classes—students majoring in Nutrition and Food Science with a concentration in Nutrition Education. We postulate that students who chose to concentrate in Nutrition Education already have a high desire to interact across cultures. Of all the constructs, desire had the highest pre scores for the beginning of the semester, so observing significant improvement was less likely.

The findings of this study offer practical suggestions for addressing the constructs deemed significant in pharmacy education: knowledge, skills, desire, sensitivity, patient focus, attitudes, and encounters [49,53]. Many of the assignments and activities can easily be incorporated into a variety of health curricula and professional training programs. Because the factor analysis results closely aligned with the constructs of the Campinha-Bacote Model and a number of classroom activities and assignments were represented for each factor, educators can use these findings to guide curriculum choices. For example, in Table 3, Factors 3 and 4 both covered encounters. In Table 4, Factors 1 and 2 each addressed knowledge and encounters, and Factors 3 and 4 both covered skill. Health educators designing cultural competence training programs could choose only one group of activities to work on a specific construct. The American College of Clinical Pharmacy White Paper advocates for a model pharmacy cultural competency curriculum in a yearly step manner [49]. Year one and two goals include cultural awareness, desire, and knowledge. Year three addresses cultural sensitivity, patient-centered focus, and skills. The fourth year focuses on encounters with diverse cultural groups. Knowledge of our factor analysis could be useful for those using this guide when planning a pharmacy curriculum.

The selection of topics, assignments, and activities were chosen based on a search of government, education, and professional organization publications and websites [54]. One such experience was Barnga, a cultural simulation card game. This simulation activity was chosen as opposed to other commonly used cultural clash games, such as BaFa’ BaFa’, because the instructions are simple, the only tools needed are decks of cards, and just one instructor is required to implement the activity. The card game worked well for our small class sizes but could easily be used with much larger groups.

Counseling models provide a framework for eliciting client perspectives regarding health beliefs, values, and practices. After understanding client viewpoints, practitioner and client can better work together to find workable solutions. Various models have been developed for health professionals working across cultures [55]. Incorporating counseling models in pharmacy education has been shown to benefit student cultural competency skill development [56]. Two culturally sensitive communication models chosen for our course were the 4 C’s of Culture and the LEARN Model. Both are easy to use, and the latter has been incorporated in several pharmacy curricula [12,56].

Because the pivotal construct of the Campinha-Bacote Model is encounters, lesson plans for our course often incorporated cross-cultural interactions with peers, media, and individuals in the community. There was also a heavy emphasis on analyzing experiences in small group discussions, journal entries, and topic papers. In order to hear a variety of perceptions, the composition of the small groups changed during each class meeting. This was a different arrangement than a cultural competency course for pharmacy students using a team-based learning approach [12]. That course also used a variety of experiences to enhance cultural competency but designed assignments best developed as teams. Evaluations of both of our courses showed significant improvement in cultural competency using the IAPCC-R assessment tool and focusing on Campinha-Bacote Model constructs. Either approach is workable and offers options for those planning pharmacy education.

After a cross-cultural experience, students were often anxious to discuss the encounter, such as after bookclub assignments. The book chosen for the book club follows the deterioration of the health of a Hmong child who had severe epilepsy. There were major clashes between the immigrant parents and the medical community. In journal entries, students reflected on their assigned readings, perceptions, and biases, and were often challenged in class discussions. Excerpts from student journal entries point to the impact of the bookclub on gaining cultural competency.

- I was fascinated by what I have read so far about the Hmong and their culture. Living in only one section of the world, I have fallen victim to general prejudices that occur when we are introduced to something completely foreign to what we are accustomed to.
- This reading has reminded me that not everyone I counsel is going to believe me when I tell them that changing their dietary habits may help them get better.
- This book was an excellent reminder of the difference in value systems from culture to culture.
- Healthcare practitioners must be cognizant of the effect of a person’s culture on any intervention. A practitioner must be aware of the patient’s needs and listen to the patient.
- This book has undoubtedly made me less ethnocentric.

Overall, Students perceived that a course providing multiple interactive activities addressing constructs of the Campinha-Bacote Model was very useful. The following contains excerpts from final student evaluations.

- …this class was extremely helpful for learning, understanding, and dealing with cultural differences.
- I was able to apply what I learned in this class clinically a few weeks ago.
- I believe this class is important for anyone in a medical field.
- I feel like this class has helped my process of becoming culturally competent.

Results of this study should be considered with the following limitations in mind. The sample size was small and diversity was limited. The greatest percentages of participants were white females. The generalizability of this study’s results should be further assessed in programs with larger class sizes. In addition, additional studies could evaluate how competency training activities and assignments interact with lecture and laboratory courses and training courses for pharmacy professionals.

## 5. Conclusions

Educational institutions have the ability to be a conduit for helping to meet cultural competency goals. In order to address health care disparities and improve health outcomes, integration of cultural competency training has been advocated for pharmacy faculty, students, and practitioners [11,49]. The findings of this curriculum evaluation indicate that learning modules focusing on experiential learning, encouraging self-evaluation and reflection, and designed to address constructs of the Campinha-Bacote Model can help improve levels of cultural competence.

The factor analysis of individual learning experiences was unique to our investigation. We not only found that overall cultural competency increased but also all class activities and assignments aligned well with predesigned Model constructs. As pharmacy educators work towards the goal of developing a standardized cultural competency curriculum, our findings can provide guidance for selection of activities and assignments that address cultural competency constructs [49].

## Figures and Tables

**Table 1:** Education experiences based on constructs of the Campinha-Bacote Model of cultural competence in the delivery of health care services for students enrolled in graduate nutrition counseling classes (*n* = 4).

Construct *	Education Experiences
Awareness	Development and presentation of cultural collage of self Barnga Simulation of cross-cultural communication [15] Project Implicit Bias Evaluation [16]
Knowledge	Terminology and definition matching activity Reading articles regarding health disparities [17,18] Cultural competency models and cross-cultural counseling algorithm lecture [19] Reading and writing quiz questions for two articles on ethnopharmacology [20,21] Cultural values contrast activity [22] Using the internet to assess and compare health disparities in various states [23] Investigation of a culture and preparation of a detailed written report Investigation and preparation of a written report on one segment of the lifespan Cultural competence assessment of a health organization, such as a health clinic [24]
Skill	Various training videos focusing on working with an interpreter and obese individuals and communicating with hearing impaired, people with disabilities, and selected religious and ethnic groups [25,26,27,28,29,30] In-class practice using PEARLS relationship-building counseling responses (Partnership, Empathy, Apologize, Respect, Legitimization, Support) [31,32] In-class experience using respondent-driven interview questions derived from Klienman’s Explanatory Model, 4 C’s of Culture, and LEARN Model [33,34,35] Conducting a guided interview across cultures using respondent-driven interview questions with a volunteer client Interview and report of a counselor who works with an identified segment of the lifespan
Encounter	Various videos including a disability dance video, *A Class Divided*, *Disability Awareness*, *Unnatural Causes*, and *Hold Your Breath* [36,37,38,39,40] Choosing Gratitude Poem [41] Book club: reading, journaling, and discussing *The Spirit Catches You and You Fall Down* [42] Various encounters with a specified cultural group including an in-person interview and a field trip to a cultural neighborhood or event In-class presentation on investigation of a culture Presentation of a poster session revealing research findings on one segment of the lifespan Interview and nutritional assessment of an individual representing one segment of the lifespan

* Cultural desire, a construct of the Campinha-Bacote Model, was not part of this table since there were no specific educational experiences addressing this construct.

**Table 2:** Evaluation of cultural competency constructs of the Campinha-Bacote Model of cultural competence in the delivery of health care services of students enrolled in a graduate nutrition counseling course (*n* = 34).

Construct	Pre	Post	F	*p*
Total	68.7 (6.9)	78.7 (6.1)	64.6	*p* < 0.001
Awareness	14.2 (1.7)	15.9 (1.8)	22.13	*p* < 0.001
Knowledge	11.6 (2.1)	15.1 (1.7)	67.2	*p* < 0.001
Skill	12.9 (1.9)	15.9 (1.6)	89.2	*p* < 0.001
Encounter	13.1 (1.7)	14.3 (1.5)	15.1	*p* < 0.001
Desire	16.8 (2.1)	17.4 (1.8)	3.05	*p* = 0.09

**Table 3:** Factor analysis of class lectures and assignments designed to improve cultural competence of students enrolled in a graduate nutrition counseling course.

Factor Group	Individual Assignment	Factor Analysis Commonality
Factor 1 (Knowledge)	Organization Cultural Competence Assessment Report	0.686
	Cross-Cultural Counseling Algorithm	0.837
	Cultural Competency Models	0.736
	LEARN Counseling Model	0.801
Factor 2 (Skill)	Cultural Interview	0.734
	Cultural Interview Report	0.773
	Cultural Report	0.538
	Cultural Oral Report	0.834
Factor 3 (Encounter)	*The Spirit Catches You and You Fall Down:* Read	0.855
	*The Spirit Catches You and You Fall Down:* Journal	0.574
	*The Spirit Catches You and You Fall Down:* Discussion	0.695
	Lifespan Poster	0.548
Factor 4 (Encounter)	Lifespan Report	0.876
	Lifespan Interview Report	0.955
Factor 5 (Awareness)	Collage	0.910
**TOTAL**	*All 5 Factors*	74.6%

**Table 4:** Factor analyses of class videos and activities designed to improve cultural competence of students enrolled in a graduate nutrition counseling course.

Factor Group	Educational Experience	Factor Analysis Communality
Factor 1 (Encounter) (Knowledge)	Disability Dance Video	0.805
	Disability Awareness Video	0.620
	Cultural Values Contrast Activity	0.754
	Ethnopharmacology Activity	0.800
Factor 2 (Encounter) (Knowledge)	Hold Your Breath Video	0.457
	Multicultural Health Video Review	0.936
	Weight Bias Video	0.740
	Internet Reading on Race and Ethnicity	0.638
	Kleinman/Respondent-Driven Interview Questions	0.564
Factor 3 (Skill)	Alzheimer’s Video	0.953
	Cross-Cultural Communication Videos	0.951
Factor 4 (Skill)	A Class Divided Simulation Video	0.711
	PEARLS: Relationship-Building Responses	0.907
	Kleinman/Respondent-Driven Interview Questions	0.537
Factor 5 (Encounter) (Skill) (Knowledge)	Unnatural Causes Video	0.872
	10 Commandments of Communicating Video	0.715
	Cultural Term Matching	0.662
Factor 6 (Awareness)	Barnga Simulation	0.875
**TOTAL**	*All 6 Factors*	83.2%

## Supplementary Materials

The following are available online at http://www.mdpi.com/2226-4787/6/2/48/s1. Table S1: Pre and post cultural competency construct data for individual students enrolled in a graduate nutrition counseling course.
