# Supplementary material for: Using a Model to Design Activity-Based Educational Experiences to Improve Cultural Competency among Graduate Students
Source: Pharmacy (Basel). 2018 Jun 1;6(2):48. doi: 10.3390/pharmacy6020048 (PMC6025602; doi:10.3390/pharmacy6020048)
Supplement: Supplementary file 1 [file pharmacy-06-00048-s001.pdf]

Table S1. Pre and Post- Cultural Competency Construct Data for Individual Students enrolled in Students Enrolled in a Graduate Nutrition Counseling Course.

	**Awareness**		**Knowledge**		**Skill**		**Encounter**		**Desire**	
Students	Pre	Post	Pre	Post	Pre	Post	Pre	Post	Pre	Post
1	15	17	14	14	12	17	11	13	13	14
2	19	20	13	15	14	19	11	12	16	16
3	15	17	11	11	9	14	9	11	10	12
4	15	16	11	16	12	16	11	11	14	15
5	12	16	12	13	12	15	10	12	14	12
6	15	12	12	14	17	16	12	11	15	12
7	14	16	12	18	13	16	11	13	14	14
8	14	17	13	17	15	18	14	14	14	16
9	12	17	11	15	11	15	11	12	13	15
10	16	14	9	14	11	15	10	11	10	14
11	13	17	8	15	12	15	10	13	12	16
12	10	15	6	15	9	13	8	10	9	11
13	15	16	11	15	13	17	11	14	13	15
14	13	17	12	19	12	17	9	14	14	16
15	14	16	14	14	15	17	10	10	13	15
16	17	13	13	17	15	17	13	12	15	13
17	16	15	12	14	13	15	10	10	13	9
18	13	18	12	16	11	13	9	9	15	13
19	13	16	10	17	10	16	10	12	12	16
20	15	15	14	14	15	15	12	14	16	13
21	15	16	14	17	15	18	12	13	16	16
22	13	16	12	17	11	18	8	14	14	15
23	16	17	11	15	15	18	13	13	15	13
24	14	15	10	14	11	16	10	11	12	13
25	13	14	17	16	14	16	12	12	12	12
26	15	18	12	15	15	17	12	12	16	12
27	15	16	12	15	12	17	9	12	9	15
28	13	19	10	14	16	18	13	15	16	16
29	17	18	14	16	13	15	13	14	13	12
30	14	16	9	13	12	14	13	12	14	14
31	14	16	9	15	14	15	10	10	13	13
32	14	15	11	16	13	15	12	14	16	15
33	11	11	13	13	13	13	10	12	14	12
34	14	14	12	13	13	13	12	10	13	13
